# Superior Effects of High-Intensity Interval vs. Moderate-Intensity Continuous Training on Endothelial Function and Cardiorespiratory Fitness in Patients With Type 1 Diabetes: A Randomized Controlled Trial

**DOI:** 10.3389/fphys.2019.00450

**Published:** 2019-04-24

**Authors:** Winston Boff, Antonio M. da Silva, Juliano B. Farinha, Josianne Rodrigues-Krause, Alvaro Reischak-Oliveira, Balduino Tschiedel, Marcia Puñales, Marcello C. Bertoluci

**Affiliations:** ^1^Post-Graduate Program in Medical Sciences, Internal Medicine Department, School of Medicine, Universidade Federal do Rio Grande do Sul (UFRGS), Porto Alegre, Brazil; ^2^Department of Physiotherapy and Rehabilitation, Universidade Federal de Santa Maria (UFSM), Santa Maria, Brazil; ^3^Exercise Research Laboratory, School of Physical Education, UFRGS, Porto Alegre, Brazil; ^4^Institute for Children with Diabetes, Hospital Criança Conceição, Grupo Hospitalar Conceição, Ministry of Health, Porto Alegre, Brazil; ^5^Endocrinology Unit, Hospital de Clínicas de Porto Alegre (HCPA), Porto Alegre, Brazil

**Keywords:** high-intensity interval training, endothelium, diabetes mellitus, type 1, flow-mediated dilation, microvascular complications

## Abstract

This study aimed to compare the effect of high-intensity interval training (HIIT) with moderate-intensity continuous training (MCT) on endothelial function, oxidative stress and clinical fitness in patients with type 1 diabetes. Thirty-six type 1 diabetic patients (mean age 23.5 ± 6 years) were randomized into 3 groups: HIIT, MCT, and a non-exercising group (CON). Exercise was performed in a stationary cycle ergometers during 40 min, 3 times/week, for 8 weeks at 50–85% maximal heart rate (HR_max_) in HIIT and 50% HR_max_ in MCT. Endothelial function was measured by flow-mediated dilation (FMD) [endothelium-dependent vasodilation (EDVD)], and smooth-muscle function by nitroglycerin-mediated dilation [endothelium-independent vasodilation (EIVD)]. Peak oxygen consumption (VO_2peak_) and oxidative stress markers were determined before and after training. Endothelial dysfunction was defined as an increase < 8% in vascular diameter after cuff release. The trial is registered at ClinicalTrials.gov, identifier: NCT03451201. Twenty-seven patients completed the 8-week protocol, 9 in each group (3 random dropouts per group). Mean baseline EDVD was similar in all groups. After training, mean absolute EDVD response improved from baseline in HIIT: + 5.5 ± 5.4%, (*P* = 0.0059), but remained unchanged in MCT: 0.2 ± 4.1% (*P* = 0.8593) and in CON: −2.6 ± 6.4% (*P* = 0.2635). EDVD increase was greater in HIIT vs. MCT (*P* = 0.0074) and CON (*P* = 0.0042) (ANOVA with Bonferroni). Baseline VO_2peak_ was similar in all groups (*P* = 0.96). VO_2peak_ increased 17.6% from baseline after HIIT (*P* = 0.0001), but only 3% after MCT (*P* = 0.055); no change was detected in CON (*P* = 0.63). EIVD was unchanged in all groups (*P* = 0.18). Glycemic control was similar in all groups. In patients with type 1 diabetes without microvascular complications, 8-week HIIT produced greater improvement in endothelial function and physical fitness than MCT at a similar glycemic control.

## Introduction

Micro- and macrovascular complications are the main causes of morbidity and mortality in patients with type 1 diabetes (Nathan et al., 1993; American Diabetes Association, 1999). Endothelial dysfunction is supposed to precede atherosclerosis and microvascular disease (Bertoluci et al., 2015). The natural course of endothelial dysfunction in type 1 diabetes is unknown, but is related to chronic hyperglycemia, oxidative stress and subclinical endothelial inflammation, leading to accelerated development of atherosclerosis (Kannel and McGee, 1979; Stehouwer et al., 2002; Schram et al., 2003). We previously demonstrated that long-term poor glycemic control is associated with endothelial dysfunction development in recently diagnosed adolescents with type 1 diabetes (Ce et al., 2011). When poor glycemic control occurs in the first few years after type 1 diabetes onset, there is a greater impact of endothelial dysfunction, indicating an effect of metabolic memory (Ce et al., 2011).

Exercise training is known to improve endothelial dysfunction. In children and adolescents with type 1 diabetes, 30 min of aerobic training for 18 weeks significantly increased flow-mediated dilation (FMD) (Seeger et al., 2011). In adults with type 1 diabetes, moderate-intensity continuous training (MCT) significantly increased FMD, after 2 months of training (Fuchsjäger-Mayrl et al., 2002). In a cross-sectional study, children and adolescents with type 1 diabetes performing more than 60 min of daily moderate to vigorous exercise had greater FMD than sedentary patients (Trigona et al., 2010). Improvements in endothelium-dependent vasodilator response is also seen in type 2 diabetes without coronary artery disease, when patients are subjected to combined aerobic and resistance training (Maiorana et al., 2001).

Intensity changes during exercise seems to be an important determinant of effects on endothelial function. Studies in different populations, including type 2 diabetes, arterial hypertension, heart failure, obesity, and metabolic syndrome have demonstrated that high-intensity interval training (HIIT) (i.e., high-intensity efforts interspersed with recovery period at lower intensity) can increase endothelium-dependent dilation more effectively than traditional MCT (Wisløff et al., 2007; Schjerve et al., 2008; Tjønna et al., 2008; Molmen-Hansen et al., 2012; Mitranun et al., 2014). In addition, HIIT is associated with greater improvement in physical fitness performance (VO_2 max_) than MCT in short-term studies. A meta-analysis involving 10 studies demonstrated that HIIT exercise provided a better physical conditioning compared to MCT in subjects with established cardiovascular disease, metabolic syndrome and obesity (Weston et al., 2014). Another recent meta-analysis found that HIIT was better than MCT in increasing VO_2 max_ in type 2 diabetes (De Nardi et al., 2018).

So far, HIIT has not been tested against MCT in patients with type 1 diabetes. Our hypothesis was that if the patient is exposed to a greater exercise intensity such as in HIIT, FMD and cardiorespiratory fitness will increase more than in MCT. Therefore, the main objective of this randomized controlled trial was to compare the effects of 8-week HIIT and MCT on endothelial function, assessed by FMD, and cardiorespiratory fitness in patients with type 1 diabetes.

## Materials and Methods

### Design

**FIGURE 1:**
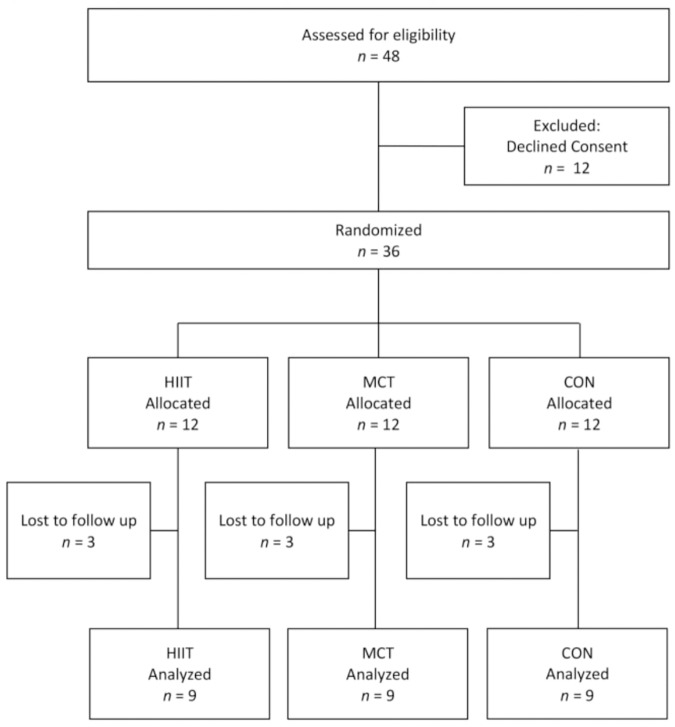
Flow diagram of inclusion of patients in the study.

A randomized, parallel-group clinical trial with 3 arms and a 1:1:1 allocation ratio. We decided to include a non-exercising group in order to control the influence of blood glucose changes in FMD. The eligibility criteria is shown in Figure 1. No changes were made to the methods after trial commencement.

### Eligibility Criteria

We searched for patients with type 1 diabetes above 18 years of age attending at the Institute for Children with Diabetes (ICD), who were included in ICD database from January 1, 2015, to January 1, 2016.

We recruited subjects of both genders, regularly attending clinic visits, who were physically inactive or not involved in exercise training programs in the previous 6 months and were interested in starting an exercise training program. We excluded smokers, pregnant women, patients with known co-morbidities not related to diabetes, patients taking drugs other than insulin and those who presented with severe diabetes-related complications, such as: loss of renal function (serum creatinine above 132.60 μmol/L), moderate to severe retinopathy or blindness, suspected or confirmed coronary artery disease, severe peripheral neuropathy, foot ulcers, or history of foot ulcers and any suspected or confirmed clinical autonomic neuropathy. Patients who met the eligibility criteria were invited to visit the research center.

### Intervention

The intervention group was submitted to the HIIT protocol. We included a moderate continuous exercise group and a non-exercising control group (CON). Training sessions were performed in the exercise training center under supervision of part of the team in the afternoon period.

As a general recommendation patients in HIIT and MCT groups exercised on cycle ergometer 3 times a week for 8 weeks. Heart rate was monitored during the whole exercise sessions using heart rate monitors (Polar^®^ FT4, Polar Electro Oy, Kempele, Finland). All exercise sessions were supervised and adherence was monitored by group. Only individuals with more than 70% of compliance were analyzed. Capillary blood glucose was measured every 5 min during all exercise training sessions and oral 10 g glucose gels were given whenever blood glucose was ≤ 5.55 mmol/L and a 20% decrease in insulin basal dose was recommended to all patients in the morning of every training day to minimize the risk of hypoglycemia. In addition, patients were recommended not to exercise at the peak of insulin action.

### High-Intensity Interval Training (HIIT) Protocol

High-intensity interval training protocol was divided into 3 phases according to Mitranun et al. (2014) phase 1: weeks 1–2, phase 2: weeks 3–4, phase 3: weeks 5–8. In phase 1, participants warmed up for 5 min, increasing intensity gradually to reach 50% of maximal heart rate. It was maintained for 20 min and then followed by a recovery period of 5 min. In phase 2, there was a 5-min warm up to reach 50% maximum heart rate (HR_max_), and it was followed by 1-minute sprint at 80% HR_max_, then slowing down intervals at 50% HR_max_ for 5 min. This procedure was repeated three more times and then followed by a recovery phase of 5 min.

In phase 3, the protocol was longer and more intense. After a 5-minute warm-up to 60% HR_max_, patients performed six 1-minute sprints at 85% HR_max_, followed by 4-minute slow-down intervals at 50%. The whole session lasted 40 min. At the end of each 1 min sprint, exercise intensity was also assessed through Borg RPE scale (rate of perceived exertion), which was independent from the heart rate. The participants of HIIT were rated as 7–8 as they informed a really hard activity, as they could speak short sentences, but could not hold a conversation.

### Moderate-Intensity Continuous Training (MCT) Protocol

Moderate-intensity continuous training protocol was also divided into 3 phases as described previously (12). In phase 1, training was identical as in HIIT. In phase 2, participants exercised to attain 50% HR_max_ in 5 min and then increased intensity to 60% HR_max_ for 20 min, ending with 5 min to recover, totaling 30 min. In phase 3, participants attained 50% HR_max_ in 5 min and exercised constantly at 65% HR_max_ for 30 min, recovering in 5 min and totaling 40 min.

### Non-exercising Control Group (CON)

Control patients were only asked to follow general lifestyle recommendations, including to walk at least three times a week for a minimum of 30 min. This group was not supervised. No other exercise specifications were given.

### Primary Outcome

#### Flow-Mediated Dilation (FMD)

We pre-specified the difference between post- and pre-training percentage FMD as the primary outcome. FMD was determined as follows. Within 2 weeks of the first visit, patients were assessed for pre-training endothelial function through brachial artery ultrasound in the left arm. The examination was performed by an experienced member, blinded to the results of the study, using the technique according to Corretti et al. (2002), which was previously described by our group (Ce et al., 2011). Tests were performed in the non-invasive cardiovascular methods unit of Hospital de Clínicas de Porto Alegre (HCPA). Briefly, patients were studied in the morning, after the usual dose of basal insulin and a 200-kcal low-fat standard meal for breakfast. Arterial blood pressure was measured by the auscultatory technique, using an aneroid sphygmomanometer, at room temperature (22–24°C). All measurements were performed using-high resolution ultrasound equipment (EnVisor CHD, Philips, Bothell, WA, United States) with a high-frequency transducer (3–12 MHz, L12-3 Philips) to obtain longitudinal images of the brachial artery. Post-training FMD evaluation and VO_2 max_ determination were assessed in a maximum of a week after the last training period.

The ultrasound images were obtained with two-dimensional mode, color and spectral Doppler. The simultaneous electrocardiography (ECG) was recorded. To minimize operational errors, both transducer and arm positions were maintained throughout the procedure. Images were recorded with the patients at rest for 30 min. Endothelium-dependent vasodilation (EDVD) and endothelium-independent vasodilation (EIVD) were determined, respectively, by FMD and nitroglycerin vasodilation. Measurements were done at multiple vascular sites using the measurement system of the same equipment. Arterial diameter measurements were done off-line, at the end of diastole, at the peak of the *R* wave in the ECG.

After recording of baseline images, the brachial artery was occluded for 5 min with a pressure cuff positioned on the arm and inflated to 50 mmHg above systolic blood pressure for 4 min. The EDVD response was recorded between 45 and 60 s after cuff release. After 10 min resting, baseline images were repeated and then 0.4 mg of sublingual nitroglycerin spray (Natispray Trinitrine, Procter & Gamble Pharmaceuticals, Paris-Cochin, France) was used to evaluate EIVD 4 min after the spray. EDVD and EIVD were expressed as percent change in brachial artery diameter before and after cuff release or nitroglycerin administration, respectively. Endothelial dysfunction was considered when EDVD was less than 8% in relation to baseline (Gaenzer et al., 2001; Gokce et al., 2003). Smooth muscle dysfunction was considered by the same criteria after nitrate use for EIVD (Gaenzer et al., 2001; Gokce et al., 2003).

### Secondary Outcome

#### Maximal Oxygen Consumption (VO_2peak_)

We pre-specified the difference between post- and pre-training VO_2peak_ values during a maximum load test. Briefly, all patients were submitted to cardiorespiratory fitness assessment one day before starting the training protocols, which was repeated within 48 h after the last training session was completed. An incremental maximal cycle ergometer (Cybex, Medway, United States) test was conducted to determine peak oxygen uptake (baseline VO_2peak_), using the breath-by-breath method in an open circuit spirometry (Quark CPET, Cosmed, Rome, Italy). After a 3-minute warm-up period, cycling at 50 W, the workload was increased by 25 W every minute until fatigue (Moser et al., 2015). VO_2peak_ was defined as the highest mean value achieved within the last 15 s prior to exhaustion.

### Sample Size Calculation

The sample size, using FMD as the primary outcome, was calculated according to the study of Mitranun et al. (2014) in type 2 diabetes, in which the effect size between post- and pre-training values of FMD was 0.49. Considering a standard deviation of 3.37%, α = 0.05 and β = 0.8, the minimal number of patients in each group was 12.

### Randomization

The randomization process was in blocks, according to FMD results before training. Baseline FMD results were ranked in decreasing order in blocks of three, so that the first three patients with corresponding higher FMD results formed the first block, in a 1-2-3 sequence, respectively, Group 1 = HIIT, Group 2 = MCT, Group 3 = CON. The following block followed an inverse sequence (3-2-1) and then consecutively. This process was performed by a collaborator outside of the study and was concealed by using numbered sealed envelopes. This process ensured that baseline FMD was similar between the three groups before training intervention.

For technical reasons, the intervention was not double-blinded, since sedentary control patients knew that they would not exercise. However, the investigator responsible for FMD determinations was blinded for the rest of the study. All further evaluations were performed before and after the exercise training interventions by the same investigators.

### Biochemical Assays

Blood and urine samples were collected after 12 h fasting. Patients were asked to avoid exercise in the 48 h before blood and urine collection. Blood samples were routinely centrifuged for 15 min and serum and plasma were stored at −80°C. HbA1c was determined by immunoturbidimetry (Certified Self-Analysis of the National Glycohemoglobin Standardization Program-Cobas Integra 400, Roche, Basel, Switzerland). Plasma glucose was evaluated by the glucose-peroxidase method using enzymatic colorimetric reactions. Serum total cholesterol, high-density lipoprotein cholesterol (HDL-c) and triglyceride concentrations were also measured by the colorimetric enzyme method (Modular, Roche, Mannheim, Germany). LDLc was estimated by the Friedewald equation. Creatinine was measured by the method of Jaffe (Modular; Roche) and high-sensitivity C-reactive protein (hs-CRP) by nephelometry (BN II; Dade-Behring, Deerfield, IL, United States). Albuminuria was determined in a single urine sample obtained in morning using the immunoturbidimetric method: Url-Pack Bayer^®^ MAlb Kit, Cobas Mira^®^ Roche (AlbUCobas) (Sacks et al., 2011).

### Oxidative Stress Parameters

Total thiol group concentrations (T-SH) were assessed by reaction with [5,5′-dithiobis (2-nitrobenzoic acid); DTNB] (Ellman, 1959), and reading at 412 nm. Levels of plasma thiobarbituric acid-reactive substances (TBARS) were evaluated as previously described (Ohkawa et al., 1979), determined spectrophotometrically at 532 nm.

### Statistics

Data distribution was evaluated by the Shapiro-Wilk test. ANOVA with Bonferroni/Dunn post-test was used to study FMD, NTG, and VO_2peak_. The differences between pre and post values for EDVD, EIVD, and VO_2peak_ were referred to as DELTA. ANOVA with Bonferroni was used to make comparisons of DELTA between groups. The chi-square test was used for qualitative variables. Pearson’s correlation coefficient was used to study the association between VO_2peak_ and FMD. Statistical tests were performed with the standard software package Statistical Analysis System (SAS) version QC (GraphPad, United States) and StatView (Abacus, United States).

### Ethics Statement

This clinical trial was registered at ClinicalTrials.gov Identifier: NCT03451201. This study protocol was approved by the HCPA ethics board, and the reported investigations were carried out in accordance with the principles of the Declaration of Helsinki. All participants provided oral and written consent prior to inclusion in the study. Those who agreed to participate were registered for further evaluation at HCPA and School of Physical Education, Physiotherapy and Dance (ESEFID).

## Results

The study randomized 36 patients with type 1 diabetes. Before the beginning of training, 9 individuals dropped out, 6 due to health problems not related to the study and 3 canceled consent for personal reasons. At the end of the study, 27 patients completed the study, 9 in each group (Figure 1). Only completers were analyzed. There were 3 random dropouts in each group that occurred immediately before the beginning of training period. All patients were analyzed in their original randomized groups. No interim analysis was performed. Patients were recruited from January 2015 to January 2016. The last follow-up visit was in May 2016. The trial ended due to the end of the protocol.

Baseline clinical and biochemical characteristics of patients are shown in Table 1. At baseline, the HIIT group showed slightly lower systolic and diastolic blood pressure values than the other groups. All other variables were similar between groups.

**Table 1:** Baseline characteristics of patients.

	HIIT (*n* = 9)	MCT (*n* = 9)	CON (*n* = 9)
Age (year)	26.1 ± 7.8	23.7 ± 5.8	20.8 ± 2.6
Male/Female	3/6	5/4	4/5
Duration of type 1 diabetes (years)	9.1 ± 2.9	10.4 ± 2.8	9.7 ± 2.7
Total daily insulin dose (Ul/kg)	0.48 ± 0.09	0.56 ± 0.22	0.47 ± 0.11
BMI (kg/m^2^)	23.2 ± 2.4	24.1 ± 2.0	22.7 ± 2.6
Systolic BP (mmHg)	108.3 ± 7.9^ab^	120.5 ± 8.8	116.5 ± 7.5
Diastolic BP (mmHg)	71.1 ± 8.2^c^	78.8 ± 7.8	79.6 ± 6.5
Fasting plasma glucose (mmol/L)	11.49 ± 4.05	8.66 ± 2.94	11.32 ± 6.16
HbA1c (%)	8.2 ± 1.3	8.4 ± 0.9	8.8 ± 2.3
Total cholesterol (mmol/L)	4.77 ± 0.77	4.57 ± 0.84	5.33 ± 1.73
LDL cholesterol (mmol/L)	2.87 ± 0.70	2.31 ± 0.57	3.16 ± 1.37
HDL-c (mmol/L)	1.53 ± 0.31	1.47 ± 0.63	1.57 ± 0.38
Triglycerides (mmol/L)	0.78 ± 0.34	1.70 ± 0.21	1.29 ± 0.87
Serum creatinine (μmol/L)	51.85 ± 9.91	59.48 ± 9.15	50.33 ± 11.44
Mean UAC (mg/L)	12.6 (3.0–41.0)	30.4 (3.3–184)	30.5 (3.0–142)
Microalbuminuria (%)	1/9 (11.1)	2/9 (22.2)	2/9 (22.2)
Endothelial dysfunction (%)	5/9 (55.5)	7/9 (77.7)	6/9 (66.6)

*BMI, body mass index; BP, blood pressure; HbAlc, glycated hemoglobin; HDL-c, high-density lipoprotein cholesterol; UAC, urinary albumin concentration. Microalbuminuria was defined as UAC > 30 mg/g. Data are expressed as mean ± standard deviation, except for UAC.*

Changes in metabolic, oxidative stress, endothelial function, and cardiovascular parameters between groups before and after training are shown in Tables 2, 3. Lipid profile, urinary albumin excretion, hs-CRP and oxidative stress measures did not differ between groups before and after training.

**Table 2:** Metabolic parameters and oxidative stress before (PRE) and after (POST) training and POST-PRE difference (Δ) in each group.

	HIIT (*n* = 9)			MCT (*n* = 9)			CON (*n* = 9)		
Variables	PRE	POST	Δ	PRE	POST	Δ	PRE	POST	Δ
Metabolic									
Weight (kg)	64.1 ± 7.3	61.7 ± 9.3	−2.36 ± 4.6	71.4 ± 11.6	70.7 ± 10.9	−0.7 ± 2.0	65.6 ± 9.5	63.3 ± 6.5	0.03 ± 0.9
FBG (mmol/L)	11.53 ± 4.0	11.44 ± 6.12	−0.08 ± 5.7	8.67 ± 2.96	9.70 ± 3.15	1.02 ± 4.17	11.34 ± 6.20	11.73 ± 6.95	−0.06 ± 9.20
HbA1c (%)	8.2 ± 1.3	8.0 ± 1.0	−0.2 ± 0.6	8.4 ± 0.9	8.1 ± 0.9	−0.3 ± 0.3	8.8 ± 2.3	9.2 ± 2.4	0.4 ± 0.8
TC (mmol/L)	4.77 ± 0.77	4.69 ± 0.93	−0.08 ± 0.57	4.56 ± 0.85	4.04 ± 1.37	−0.01 ± 1.45	5.34 ± 1.74	5.54 ± 2.05	0.21 ± 0.98
LDL-c (mmol/L)	2.87 ± 0.70	2.80 ± 0.80	0.09 ± 0.56	2.31 ± 0.57	1.81 ± 1.24	−0.51 ± 1.32	3.16 ± 1.37	3.16 ± 1.40	0.00 ± 0.75
HDL-c (mmol/L)	1.53 ± 0.31	1.54 ± 0.42	0.01 ± 0.18	1.47 ± 0.63	1.37 ± 0.51	−0.08 ± 0.20	1.57 ± 0.38	1.62 ± 0.55	0.05 ± 0.27
TG (mmol/L)	0.78 ± 0.34	0.80 ± 0.28	0.02 ± 0.25	1.70 ± 0.21	1.86 ± 0.16	0.16 ± 0.92	1.29 ± 0.87	1.64 ± 1.53	0.32 ± 0.71
hs-CRP (nmol/L)	18.1 ± 20.9	19.0 ± 15.2	0.9 ± 9.5	31.4 ± 23.8	37.1 ± 31.4	5.7 ± 24.7	47.6 ± 43.8	102.8 ± 147.6	55.2 ± 153.3
Oxidative Stress									
TBARS (μM MDA/L)	2.00 ± 1.41	2.31 ± 1.60	0.31 ± 0.45	2.18 ± 0.59	2.68 ± 1.54	0.49 ± 1.72	2.40 ± 0.76	2.35 ± 1.67	−0.04 ± 2.63
T-SH (nmol/mg GSH)	79.5 ± 15.2	88.3 ± 12.1	8.77 ± 17.2	95.0 ± 28.3	96.2 ± 14.0	1.2 ± 25.01	95.3 ± 29.7	91.3 ± 21.7	−4.0 ± 32.5

*CON, non-exercising controls; FBG, fasting blood glucose; HbAlc, Hemoglobin Alc; HDL-c, high-density lipoprotein cholesterol; HIIT, high intensity interval training; hs-CRP, high sensitivity C-reactive protein; LDL-c, low density lipoprotein cholesterol; MCT, moderate continuous training; TBARS, plasma thiobarbituric acid-reactive substances; TC, total cholesterol; TG, triglycerides; T-SH, total thiol group concentrations; VC_2 max_, maximal oxygen consumption during exercise.*

**Table 3:** Endothelial function and cardiovascular parameters before (PRE) and after (POST) training and POST-PRE difference (Δ) in each group.

	HIIT (*n* = 9)			MCT (*n* = 9)			CON (*n* = 9)		
Variables	PRE	POST	Δ	PRE	POST	Δ	PRE	POST	Δ
Endothelial Dysfunction									
Mean FMD (%)	5.7 ± 5.0	11.2 ± 5.4^abc^	5.5 ± 4.4^de^	5.2 ± 3.3	5.4 ± 3.3	0.24 ± 4.0	7.6 ± 7.4	5.0 ± 3.3	−2.6 ± 6.4
% with ED	5/9 (55.5)	2/9 (22.2)^ac^	–	7/9 (77.7)	8/9 (88.8)	–	6/9 (66.6)	8/9 (88.8)	–
Mean NTG (%)	24.1 ± 7.3	22.5 ± 5.3	−1.5 ± 5.4	18.0 ± 4.2	16.3 ± 4.7	−1.7 ± 3.9	26.3 ± 6.7	18.6 ± 7.7	−4.3 ± 6.2
% with SMD	0	0	–	0	0	–	0	0	–
Cardiovascular									
Systolic BP (mmHg)	108.3 ± 7.9	116.1 ± 9.2	7.7 ± 9.3^d^	120.5 ± 8.8	118.2 ± 7.8	−1.6 ± 8.6	116.5 ± 7.5	120.5 ± 7.2	4.0 ± 5.5
Diastolic BP (mmHg)	71.1 ± 8.2	78.8 ± 8.9	7.7 ± 9.0	78.8 ± 7.8	81.6 ± 8.2	2.7 ± 8.7	79.6 ± 6.5	80.6 ± 7.6	1.0 ± 7.4
Resting HR (bpm)	76.5 ± 11.7	74.4 ± 8.7	−2.1 ± 12.5	73.2 ± 4.7	76.0 ± 8.4	2.1 ± 8.3	77.7 ± 10.5	84.1 ± 7.5	6.3 ± 8.5
Max HR_peak_ (bpm)	180.4 ± 14	189.0 ± 16	8.51 ± 13.9^c^	179.2 ± 16	178.3 ± 14.9	−0.88 5.32	183.2 ± 15	184.3 ± 16	0.55 ± 4.12
VO_2peak_ (ml/kg/min)	34 ± 6.3	40.1 ± 4.3	6.08 ± 2.58^db^	33 ± 8.2	36 ± 8.8	3.04 ± 4.03^c^	33.2 ± 10	32.7 ± 10	−0.34 ± 2.78

*% with ED, percent of patients with endothelial dysfunction; % with SMD, percent of patients with smooth muscle dysfunction; BP, blood pressure; CON, non-exercising controls; HIIT, high intensity interval training; HR, heart rate; Max HR_peak_, maximal heart rate; MCT, moderate continuous training; Mean FMD, mean flow-mediated dilation; Mean NTG, mean of nitroglycerin-mediated dilation; VO_2peak_, peak oxygen consumption. Data are expressed as mean and standard deviation. PRE, corresponds to value obtained immediately before first training session; POST, correspond to values obtained immediately after the last exercise session; Δ corresponds to the mean of difference between post and pre values. ^a^P < 0.01 vs. MCT; ^b^P < 0.01 vs. baseline-HIIT; ^c^P < 0.01 vs. CON; ^d^P < 0.05 vs. MCT; ^e^P < 0.05 vs. CON.*

**FIGURE 2:**
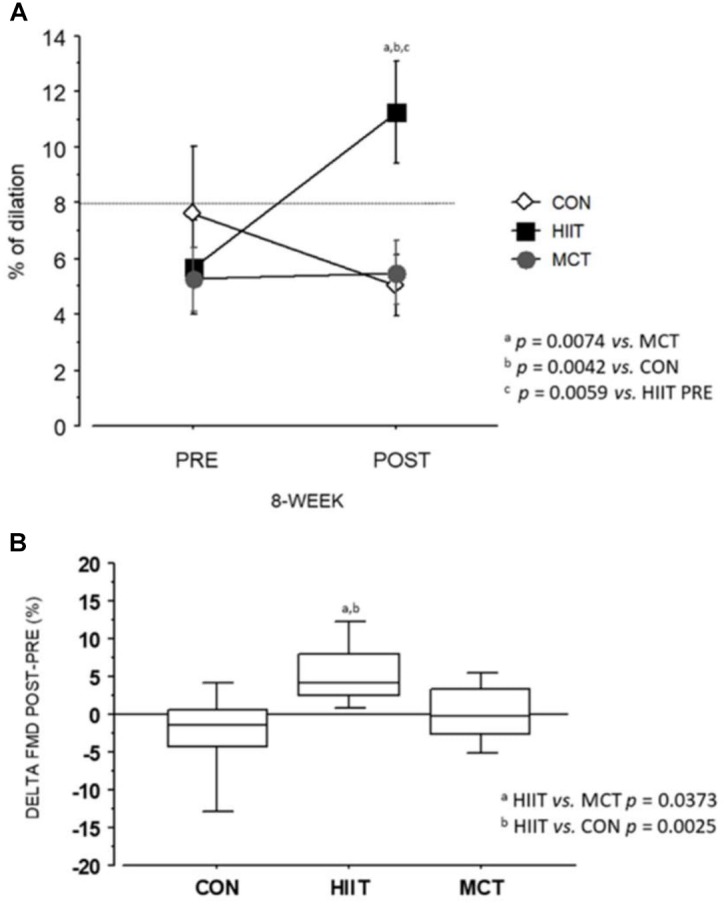
Flow Mediated dilation (FMD): **(A)** Befor and training. **(B)** Difference between post and pre-training.

At baseline, the percentage of patients with endothelial dysfunction (% with ED) was similar in all groups (*P* = 0.60), as well as the baseline mean EDVD (Table 3). After training, % with ED was significantly lower in HIIT (22.2%) vs. MCT (88.8%) (*P* = 0.044) and vs. CON 88.8% (*P* = 0.0184) (Table 3). After training, EDVD increased from baseline in HIIT (*P* = 0.0059) and was significantly greater in relation to MCT (*P* = 0.0074) and CON (*P* = 0.0042) (Figure 2). No increase in EDVD was seen in MCT or CON. EIVD was unchanged between pre- and post-training in all groups.

Although systolic blood pressure (SBP) values were in the normal range within groups before and after training, SBP increased 7.4% in HIIT (*P* = 0.0378), while it was unchanged in MCT (*P* = 0.58) and CON (*P* = 0.08). Maximal heart rate was increased in HIIT in relation to CON (*P* < 0.05). There was no difference in maximal heart rate between MCT and CON (Table 3).

VO_2peak_ was similar at baseline between groups (*P* = 0.96) and increased 17.6% from baseline after HIIT training (*P* = 0.0001) but only 3% in MCT (*P* = 0.055), with no change in CON (*P* = 0.63). There was a trend for a greater increase in VO_2peak_ after training in HIIT compared to MCT (*P* = 0.055) (Table 3).

**FIGURE 3:**
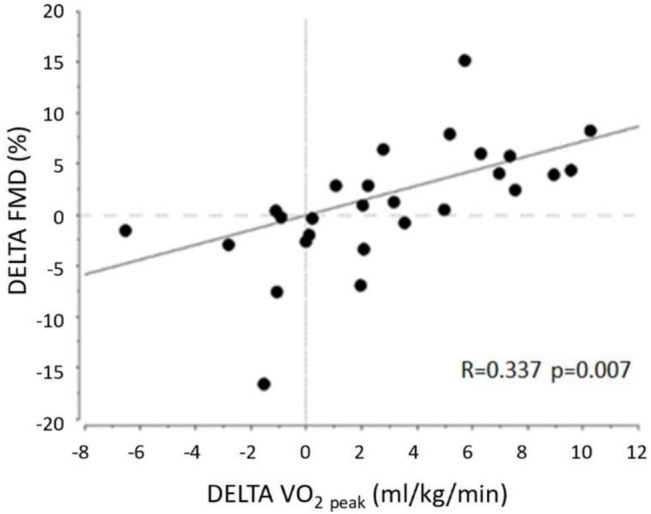
Correlation between increase in FMD and in peak Oxygen Consumption (VO_2Peak_) in all patients.

We found a positive correlation (*r* = 0.337, *P* = 0.007) between the delta of VO_2peak_ and the delta of FMD, indicating that a better cardiorespiratory fitness was associated with an improvement in endothelial function (Figure 3).

After training, HbA1c was not significantly changed compared to baseline values in any of the protocol groups. No serious hypoglycemic episodes occurred. No patient had muscular injury nor cardiovascular symptoms. All supervised exercise sessions were completed.

## Discussion

This randomized clinical trial examined the effects of HIIT in relation to MCT on endothelial function of young adults, with non-complicated type 1 diabetes. The study showed that 8 weeks of HIIT markedly improved vascular function, by increasing EDVD 2-fold from baseline, significantly more than MCT during a similar period of training. This was not dependent on improvements in glycemic control. Moreover, HIIT produced a robust improvement in physical fitness from baseline, while there was only a mild improvement in MCT. The strong positive correlation observed between the improvement in FMD and improvement in VO_2peak_ indicated that these variables are interdependent, and that changing intensity during exercise is an important determinant to improve physical fitness and vascular improvement in young patients with type 1 diabetes.

Exercise can improve endothelial function in both type 1 and type 2 diabetes when compared to non-exercising controls. Three studies have previously evaluated FMD in type 1 diabetes using different protocols. In a non-controlled study, Seeger et al. (2011) observed that, after performing 30-min sessions of aerobic training twice a week for 18 weeks, children and adolescents with type 1 diabetes showed a 65% increase in FMD, compared to non-exercising controls. Fuchsjäger-Mayrl et al., 2002 in a non-randomized trial tested a similar protocol of moderate continuous exercise training in older individuals with type 1 diabetes and observed improvements in FMD only after 4 months of training. They used continuous moderate workloads of 60–70% of VO_2 max_ for 40 min in a stationary cycle ergometer, 2–3 times a week and observed that FMD improved by more than 50%, in adults with non-complicated type 1 diabetes, while no change was observed in non-exercising individuals. In a cross-sectional study (Trigona et al., 2010) including children and adolescents with type 1 diabetes, there was an association between FMD and exercising, and endothelial function was enhanced in patients who engaged in more than 60 min/day of moderate-to-vigorous physical activity. These studies indicate that moderate continuous training may improve FMD in patients with type 1 diabetes. However, MCT may take too long to show benefits what is concerning because, in general, people with diabetes tend to exercise for short periods.

Although exercise training effects have been studied in type 1 diabetes, this is, to the best of our knowledge, the first study comparing HIIT and MCT in a head-to-head randomized clinical trial. The effect of short intervals during exercise sessions was studied, however, in type 2 diabetes. Interval and continuous exercise training were compared in an open label clinical trial in relation to microvascular reactivity. Mitranun et al. (2014) randomized 45 patients with type 2 diabetes to perform exercise sessions with similar energy expenditure, walking on a treadmill for 30 and 40 min per day for 12 weeks. They observed that both continuous and interval exercise training were effective in improving FMD from baseline, but there was a greater improvement in FMD in the group that performed intensity exercise intervals than in those who exercised in the continuous training group (37 vs. 27% increase, *P* < 0.05, respectively). In the present study, the differences in FMD caused by interval training were even more robust than those observed in type 2 diabetes by Mitranun et al. (2014). We found that there was an almost doubling of FMD from baseline in HIIT (97% increase) with no change in MCT at 8 weeks.

It is well known that acute exercise can enhance endothelial function compared to non-exercising controls in different clinical conditions. A meta-analysis indicated that all exercise modalities can enhance endothelial function (Ashor et al., 2015). Exercise can enhance endothelial function basically through four mechanisms: (1) by increasing nitric oxide (NO) bioavailability, which occurs secondarily to enhanced expression/stabilization of endothelial nitric oxide synthase enzyme (eNOS) and/or reduced inactivation/degradation of NO by free-radicals (Newcomer et al., 2011); (2) increasing expression of antioxidant enzymes, superoxide dismutase, glutathione peroxidase and catalases thus enhancing antioxidant capacity (Belardinelli et al., 2005) as well as reducing the expression of oxidant enzymes, such as nicotinamide adenine dinucleotide phosphate (NADPH) oxidase (Gielen et al., 2010); (3) reducing the expression of pro-inflammatory molecules such as interleukins, adhesion molecules (Ribeiro and Oliveira, 2010); and finally (4) increasing the number of endothelial progenitor cells (EPCs), which are important determinants of vascular endothelium regeneration and angiogenesis (Ribeiro et al., 2013).

The mechanism by which interval training could exert effects on endothelial function is still speculative. It is plausible, however, that shear stress could be increased after exercise (Dopheide et al., 2017) inducing eNOS phosphorylation (Casey et al., 2017). In a pilot trial, Dopheide et al. (2017) evaluated mean tangential wall stress (TWS) in the femoral artery, a surrogate marker of shear stress, in 40 patients with known peripheral artery disease, who were compared to healthy individuals before and after supervised exercise training. Patients were asked to walk 60 min per day, at least 3–5 days a week. The intensity was limited by claudication, and they should rest for intervals of up to 5 min, repeating the same distance at lower intensity. There was a significant increase in TWS in relation to controls, indicating that intermittent exercise training could increase shear stress. Moreover, a recent study (Casey et al., 2017) demonstrated that repeated muscle contraction can induce eNOS phosphorylation in humans by increasing arterial shear stress. Casey et al. (2017) studied seven young males who performed 20 bouts of rhythmic forearm exercise at 20% maximal (3 min each) separated by 3 min of rest, over a 2 h period. Fresh endothelial cells were then obtained 2 h after exercise. They observed that protein expression and phosphorylation of eNOS was increased. This was the first evidence in humans that muscle contraction-induced increases in conduit arterial shear could lead to *in vivo* posttranslational modification of eNOS activity in endothelial cells.

Endothelial dysfunction in type 1 diabetes is known to be caused by chronic hyperglycemia and increased oxidative stress, also worsened by early vascular rigidity (Bertoluci et al., 2015). In the present study, however, exercise training did not change oxidative stress markers such as TBARS or T-SH levels, not supporting the hypothesis that a reduction in oxidative stress was critical for short-term improvements in endothelial function.

The present study had some limitations to be considered. (1) Since we studied young patients with type 1 diabetes without established diabetic complications, extrapolating these results to a group with more advanced disease is limited. (2) Our results are limited to 8 week of training. Long-term effects of exercise on endothelial function in type 1 diabetes are unknown. (3) As our study protocol was restricted to 8 weeks, it may have limited the detection of improvements of FMD in MCT group. In the study of Fuchsjäger-Mayrl et al. (2002, which was non-randomized, 18 type 1 patients were submitted to 2–4 months training at continuous (40 min) submaximal workload (60–70%VO_2 max_) for 1 h, 2–3 times a week and were assessed through FMD at the brachial artery, a very similar protocol to that used in the MCT group in the present study. They observed that the endothelium-dependent vasodilatory response to reactive hyperemia increased significantly only after 4 months of training. This indicates that MCT may also be effective in improving FMD in type 1 diabetes, but may take a longer time. By this way, our study might be underpowered to detect such FMD changes in MCT group.

On the other hand, there were some important strengths to be considered. (1) This is the first randomized clinical trial study to compare HIIT protocol head-to-head with MCT in young adults with type 1 diabetes. We used the in-block randomization process, considering FMD, which favored to obtain very similar FMD values at baseline, without selection bias. (2) We had a very high compliance level. (3) Although we may have had included a theoretically small number of patients, we had no losses during the training protocol. All losses and all included patients were studied in their original groups, as the losses occurred before training and were similar in all groups at random (4) Finally, our FMD measurements were performed by a single highly trained blinded examiner, increasing accuracy. In the future studies, however, the use of an edge detection software system can be useful to improve the validity of the FMD measurements.

## Conclusion

In young adults with type 1 diabetes without known complications and in moderate glycemic control, HIIT proved to be superior to MCT in improving endothelial dysfunction and physical fitness during a training period of 8-weeks. The effect on endothelial function was closely related to improvement in physical fitness and did not depend on glycemic control changes. Thus, HIIT can be recommended as a useful and safe non-pharmacological alternative to improve vascular function in patients with type 1 diabetes. Long-term studies to examine the efficacy of HIIT in preventing micro- and macrovascular disease are still required.

## Ethics Statement

This clinical trial was registered at ClinicalTrials.gov Identifier: NCT03451201. This study protocol was approved by the Hospital de Clínicas de Porto Alegre (HCPA) ethics board (CAEE 54928116.0.0000.5327) and the reported investigations were carried out in accordance with the principles of the Declaration of Helsinki. All participants provided oral and written consent prior to inclusion in the study.

## Author Contributions

WB was the mentor of the study, designed, and organized the logistics at Hospital de Clínicas de Porto Alegre (HCPA), submitted the manuscript, conceived and executed the study, including the supervised exercise sessions. AdS performed all ultrasound examinations and obtained FMD data. JF and JR-K collected the data and organized the database at the Exercise Research Laboratory. AR-O implemented the study design in the Exercise Research Laboratory, supervised the exercise protocol, wrote and revised part of the manuscript. BT and MP organized the database and logistics at the Institute for Children with Diabetes (ICD) and revised the manuscript. WB designed the study, wrote and revised the manuscript. MB reviewed the manuscript and raised financial funds for publication.

## Conflict of Interest Statement

The authors declare that the research was conducted in the absence of any commercial or financial relationships that could be construed as a potential conflict of interest.
